# Bound vortex light in an emulated topological defect in photonic lattices

**DOI:** 10.1038/s41377-022-00931-4

**Published:** 2022-08-01

**Authors:** Chong Sheng, Yao Wang, Yijun Chang, Huiming Wang, Yongheng Lu, Yingyue Yang, Shining Zhu, Xianmin Jin, Hui Liu

**Affiliations:** 1 National Laboratory of Solid State Microstructures and School of Physics, Collaborative Innovation Center of Advanced Microstructures, Nanjing University, Nanjing, Jiangsu 210093 China; 2 Center for Integrated Quantum Information Technologies (IQIT), School of Physics and Astronomy and State Key Laboratory of Advanced Optical Communication Systems and Networks, Shanghai Jiao Tong University, Shanghai, 200240 China; 3 CAS Center for Excellence and Synergetic Innovation Center in Quantum Information and Quantum Physics, University of Science and Technology of China, Hefei, 230026 China

**Keywords:** Transformation optics, Integrated optics

## Abstract

Topology have prevailed in a variety of branches of physics. And topological defects in cosmology are speculated akin to dislocation or disclination in solids or liquid crystals. With the development of classical and quantum simulation, such speculative topological defects are well-emulated in a variety of condensed matter systems. Especially, the underlying theoretical foundations can be extensively applied to realize novel optical applications. Here, with the aid of transformation optics, we experimentally demonstrated bound vortex light on optical chips by simulating gauge fields of topological linear defects in cosmology through position-dependent coupling coefficients in a deformed photonic graphene. Furthermore, these types of photonic lattices inspired by topological linear defects can simultaneously generate and transport optical vortices, and even can control the orbital angular momentum of photons on integrated optical chips.

## Abstract (web summary)

Optical vortices in a topological defect of cosmic space-time.

## Graphical abstract

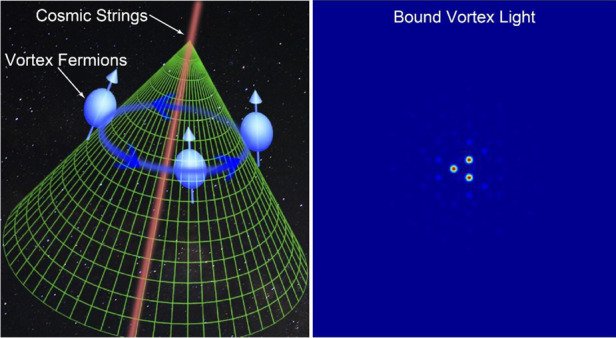

## Introduction

Photonic lattices^1^ made of single-mode coupled waveguides have become a hugely popular tool for simulating a plethora of physical phenomena, ranging from reservoir engineering^2^, demonstration of flat-band states and dispersion-less light propagation^3–6^, topological bandgaps and edge modes^7–9^, to topological pumping^10,11^ and Aharonov-Bohm cages^12–14^, etc. Intriguingly, photonic graphene, one of novel types of photonic lattices, have successfully emulated various topological phenomena in condensed matter physics, for example, photonic Floquet topological insulators using spiral waveguides^15^, Landau levels using strain lattices^16^, the unconventional edge states^17,18^, the pseudospin-mediated vortex generation^19^. Additionally, photonic lattices have also allowed for the simulation of quantum physics in general relativity^20–24^, such as fermion pair production^22–24^, hyperbolic space^25,26^ with constant negative curvature connecting to anti-de Sitter/conformal field theory correspondence.

On the other hand, a stochastic gravitational wave background^27–29^, which was possibly emitted by a cosmic-string network^27,28^ that is a topological linear defect generated in the early Universe, has attracted widespread attention in cosmology. Aside from gravitational waves, theorists have predicted some other interesting nontrivial phenomena about these cosmic topological defects, such as the Aharonov-Bohm interaction of cosmic strings^30–32^ and the spin hall effect influenced by the nontrivial deformation of the space time of cosmic strings^33^. Although these predictions cannot be directly found in astronomy up to now, the theoretical foundations behind these phenomena of general relativity have been extensively applied to condensed matter systems and other various systems. One of the salient examples is the Hawking-Unruh effect near the event horizons, which has been well-emulated using a variety of quantum architectures^34–38^.

At the same time, transformation optics^39,40^ which was inspired by general relativity has provided a new method to design artificial materials with novel optical functions. One fascinating example is invisibility cloaks^41–43^, in which light is regarded as linear parallel geodesic rays in deformed spaces. Additionally, other examples include the light trapping^44–48^ by emulating black holes, the illusion electromagnetic devices^49^, the definite electromagnetic scattering^50^ in the emulated nontrivial space and the wavefront shaping^51–53^ on curved space, etc. However, all these novel transformation optics devices were constructed by emulating space without a gauge field. Indeed, some general relativity phenomena^30–33^ involve both a gravitational field and a gauge field. And what novel optical application will be brought by the design, which simulates a gravitational field and a gauge field at the same time, is rarely experimentally reported.

Recently, there has been an increasing interest in optical vortices that have wide-ranging applications in micromanipulation^54^, free-space communications^55^, and quantum information technology^56^. The generation of optical vortices has been investigated for various optical elements, including computer-generated holograms^57^, inhomogeneous anisotropic media^58^, angular gratings inside microrings^59^, and metasurfaces^60^. However, in most of the reported works, the generated vortex states mainly radiate in free space, whereas there are few works regarding the simultaneous generation and transportation of vortex states on photonic chips. In contrast, the development of integrated optical chips requires a simple scheme to perform the simultaneous generation and transmission of optical vortices on chips. Although the doughnut-shaped waveguide^61,62^ on a photonic chip has been experimentally exploited to generate and transmit optical vortices, this type of generation has the aid of an external auxiliary waveguide. The simultaneous generation and transmission of optical vortices in the same process has rarely been reported.

In this work, we exploited a deformed photonic graphene, a novel type of photonic lattices, to map massless Dirac fermions in the presence of the gauge field of a cosmic string. We experimentally realized bound vortex light by simulating the gauge field of such a cosmic string. Our approach is to deform the photonic graphene lattice by deliberately tuning the coupling coefficient as a function of the position according to the effective Hamiltonian after considering the massless Dirac equation in the presence of such a topological linear defect. The required anisotropic velocity of Dirac fermion in the polar coordinate are obtained by the designed anisotropic and nonuniform coupling coefficient. Meanwhile, such designed coupling coefficients also shift the Dirac cone in the momentum space to yield the required gauge field. Very recently, the topological lattice defects^63^ in Weyl crystals were demonstrated to bind acoustic vortex states through a judicious “cut-and-glue” procedure. Compared to the generation of topological defects through the deficit of the physical structure, we leverage the position-dependent coupling coefficient in a photonic graphene to emulate the cosmic string. Remarkably, such photonic lattices can simultaneously generate and transport optical vortex modes nearly without diffusion, and even have an ability to manipulate the angular momentum of photons on a photonic chip.

## Results

### The mapping of the Dirac equation of cosmic stings into photonic lattices

**Fig. 1:**
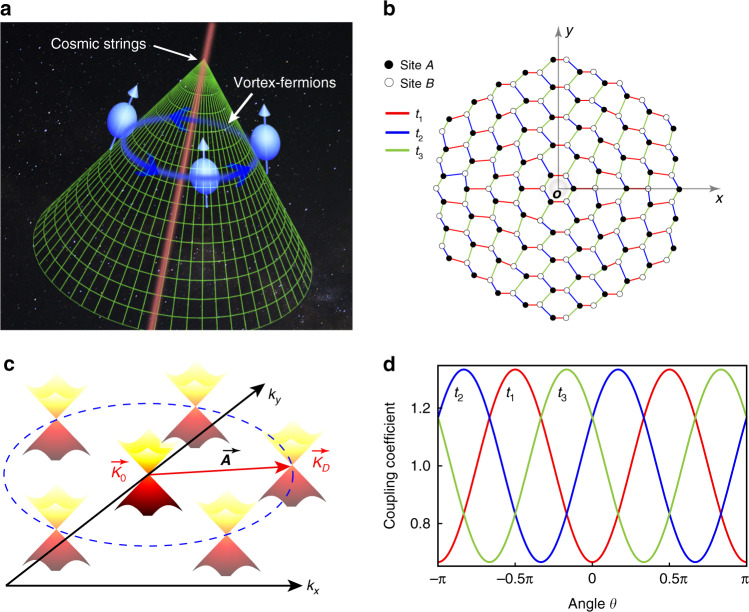
The schematic of bound vortex fermions in the gauge field of cosmic strings and the constructed photonic graphene with the position dependent coupling coefficients. **a** The depiction of a bound vortex of fermions in the gauge field of the cosmic string. **b** The schematic of the deformed photonic graphene lattice with a position-dependent coefficient to emulate the cosmic string with a gauge field. The symbol *O* is the origin center of the photonic graphene lattice where the defect was located. **c** The schematic of the movement of the Dirac cone $\vec K_D$ circling around $\vec K_0$ with the variation of the coupling coefficient; ${{{\vec{\boldsymbol A}}}}$ is the momentum shift of the Dirac cone in the deformed photonic graphene. **d** The designed coupling coefficient in a uniform photonic graphene to emulate a cosmic string with a gauge field. In the actual waveguide system, such space dependent coefficients are realized by deforming photonic graphene just as shown in **b**

To study the vortex bound Dirac fermion mode in the presence of cosmic strings, we began with the massless Dirac equation with a gauge field in the curved space as follows^64,65^:

1 $$\gamma ^u\left( {\partial _u + \Omega _u + iA_u} \right)\psi = 0$$

where *γ*^*u*^ is the Dirac matrix in a curved background, Ω_*u*_ is the spinor connection, and *A*_*u*_ is the electromagnetic gauge potential. In the following, we will focus our calculations on the cosmic-string space time at a certain plane (see Fig. 1a), and the line element is given by

2 $$ds^2 = - dt^2 + dr^2 + \alpha ^2r^2d\theta ^2$$

where $\alpha = 1 - 4G\mu /c^2$ is the density parameter that corresponds to the deficit angle, *G* is the gravitational constant, *μ* and *c* are respectively the linear mass density of the cosmic string and the speed of light. After considering the spacetime metric of cosmic strings, we obtain spinor connection $\Omega _\theta = \frac{i}{2}\left( {1 - \alpha } \right)\sigma _z$. Furthermore, considering that the cosmic string has electromagnetic gauge potential with $A_x = \eta \sin 2\theta$, $A_y = \eta \cos 2\theta$ ($\eta = - \left( {1 - \alpha } \right)/\left( {1 + \alpha } \right)$ is the amplitude of the gauge field), we obtained the Dirac fermion with anisotropic velocities for the radial and angular direction as $v_r/v_\theta = \alpha$ and the gauge field as $A_r = \eta \sin 3\theta ,A_\theta = \alpha r\eta \cos 3\theta$ (see details in the Supplementary Section I). Hence, both the anisotropy of the velocity and the gauge field for the massless Dirac fermion were decided by the mass density parameter *α* of the cosmic string. For the case of the flat space (*α* *=* 1), the massless Dirac fermion had isotropic velocity and was not subjected to a gauge field. For the case of cosmic strings with a deficit angle (*α* *<* 1), we could obtain the massless Dirac fermion with anisotropic velocity and a nonzero gauge field. Moreover, both the anisotropy of the fermion velocity and the amplitude of the gauge field increased with the increases of the deficit angle of the cosmic string.

To construct the quantum field of Dirac fermions in the presence of cosmic strings, we chose a deformed photonic graphene lattice (Fig. 1b) as a real system that was as close as possible to the required quantum field^20^. We considered that the hopping between the nearest sites was space dependent, and in general, it was characterized by three hopping parameters *t*_1_, *t*_2_, and *t*_3_. Within this nearest-neighbor tight-binding model, one could demonstrate that the effective Dirac Hamiltonian could be obtained by expanding the Hamiltonian around a corner, e.g., $\vec K_0 = \left( {0,\; \frac{{4\pi }}{{3\sqrt 3 a}}} \right)$ for the isotropic case *t*_1,2,3_=*t*_0_. However, for the anisotropic case^66^ $t_1 = t_0\left( {1 + \eta \cos 2\theta } \right)$, $t_2 = t_0\left( {1 - \eta \cos \left( {2\theta - \frac{\pi }{3}} \right)} \right)$, $t_3 = t_0\left( {1 - \eta \cos \left( {2\theta + \frac{\pi }{3}} \right)} \right)$ as shown in Fig. 1d, and one could determine that the Dirac point $\vec K_D$ was given by $\vec K_D = \vec K_0 + \vec A$ (see Fig. 1c), where $\vec A$ is the gauge field given by $A_x = \eta \sin 2\theta ,A_y = \eta \cos 2\theta$, circling around the origin of the cosmic strings (For the case that $\vec K_0$ with valley index −1 see details in the Supplementary Section II). Moreover, one could obtain an effective Dirac Hamiltonian (see details in the Supplementary Section II):

3 $$E_F = \hbar v_F^\prime i\sigma _z\vec \sigma \cdot U^{ - 1}\left( \theta \right)\left( {\begin{array}{*{20}{c}} \alpha & 0 \\ 0 & 1 \end{array}} \right)U\left( \theta \right) \cdot \vec q$$

where $v_F^\prime$ is the reduced Fermi velocity for the massless Dirac fermion, $\vec \sigma = \left( {\sigma _x,\sigma _y} \right)$ represents the non-diagonal Pauli matrices, *σ*_*z*_ is the diagonal Pauli matrix, $\vec q$ is the momentum vector, and $$U\left( \theta \right) = \left( {\begin{array}{*{20}{c}} {\cos \theta } & {\sin \theta } \\ { - \sin \theta } & {\cos \theta } \end{array}} \right)$$ is the rotation operator. Based on the effective Dirac Hamiltonian and the shift of the Dirac cone in the momentum space, the Dirac fermion conducted by the deformed photonic graphene had the anisotropic velocity of $v_r/v_\theta = \alpha$ and it was subjected to the gauge field with $A_r = \eta \sin 3\theta ,A_\theta = \alpha r\eta \cos 3\theta$, which satisfied the condition required by the quantum field of massless Dirac fermions in the presence of cosmic strings.

### The generation and transmission of vortex states in the emulated cosmic strings

To construct the coupling coefficients as required by emulating cosmic strings, we exploited the femtosecond laser direct writing technique to fabricate single-mode waveguides in borosilicate glass. Since the coupling strength drops quite steeply with the distance, only the nearest-neighbor coupling is considered. Owing to the fabrication advantages of femtosecond laser direct writing that can freely prototype waveguides as sites of lattices and precisely control coupling in three dimensions, the deformed photonic graphene (as shown in the inset of Fig. 3e) had space dependent nearest-neighbor coupling coefficients and it could emulate the cosmic string with a gauge field. Because such an evanescently coupled waveguide array rendered the evolution of photons for the Schrödinger-like equation and allowed us to directly observe the quantum dynamics, we could directly study the quantum evolution for the emulated cosmic string with a gauge field.

**Fig. 2:**
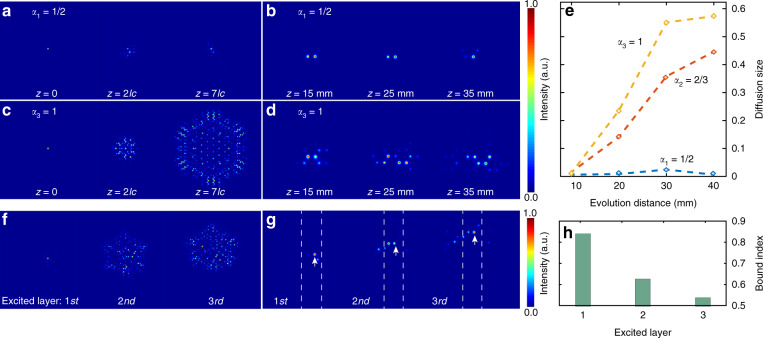
The generation and transmission of bound optical vortices in deformed photonic graphene with the injection of photons into a single waveguide. **a** and **b** are respectively the theoretical calculated results and experimental results with the excitation of a single waveguide in the innermost ring layer around the cosmic string. **c** and **d** are respectively the theoretical calculated results and experimental results with the excitation of a single waveguide in the uniform graphene. **e** is the experimental diffusion size as the evolution distance for different density parameters of the cosmic strings. **f** and **g** are respectively the theoretical calculated results and experimental results with the excitation of a single waveguide on different ring layers after same propagational distance. The white arrows indicated the waveguide into which the input light beam was launched in g. In f the propagational distance is *z* =5*l*_*c*_ (*l*_*c*_ is the coupling distance), in g the propagational distance is *z* = 25 mm. And the sites between the dashed lines in g were used to define the bound index that is the proportion of the photon distribution into such a zone among all sites. **h** is the bound index as the excitation of a single waveguide on different ring layers. The theoretical calculation was carried out in the graphene lattice whose size was 1302 waveguide sites. Whereas considering the limitation of experimental fabrication, the experimental results were determined using a graphene lattice with 150 waveguide sites

In experiments, we successfully observed a vortex bound fermion mode in the deformed photonic graphene. Figure 2a, b clearly exhibit the confined photons evolving around the cosmic string and propagating with different distances after the photons were injected into a single waveguide site (the details of such bound states from the tight-binding model in Supplementary Section III, IV). Although a small amount of the energy radiated out, the energy that circled around the cosmic string was dominant. As expected, the confinement of the injected photons into different sublattice sites of the innermost layer exhibited the same behavior (see Fig. S3 in the Supplementary). In comparison, there were only radiating modes in flat space (analogous to unperturbed photonic graphene) and the energy could not be trapped any more, as shown in Fig. 2c and d. To quantify the diffusion size of the bound vortices for the different gauge fields of the emulated cosmic string, we show the proportion of the photon distribution among the sites outside the innermost layer in Fig. 2e. The photons were strongly localized around the cosmic string with the density parameter *α*_1_ = 1/2, slightly localized for the case with the density parameter *α*_2_ = 2/3, and widely diffused in flat space (*α*_3_ = 1). These phenomena distinctly clarified the fact that the emulated cosmic string with a larger deficit angle had a larger amplitude of the gauge field, leading to a higher capability to confine photons into a vortex. Although the losses and imperfections induced by fabrication and materials were unavoidable in experiments, the experimental results were agreement with the theoretical calculation.

Furthermore, the magnetic field in the presence of the cosmic string had the form of *B*_*z*_ ∝ 1/*r*, and the magnetic field strength decayed with the distance away from the cosmic string (see details in the Supplementary Section I). Hence, the confinement ability of the photons decreased with the distance away from the cosmic string, as shown in Fig. 2f. In the experiment, we injected the photons into a single site in the first layer, second layer, and third layer along the vertical direction, and as theoretically expected, the photons gradually diffused (see Fig. 2g). We defined the bound index as the proportion of the photon distribution into the innermost layer among all sites (the range between white dashed lines in Fig. 2g). The result of the bound index shown in Fig. 2h accurately reflected the decay of the magnetic field strength with the distance away from the cosmic string.

### Bound vortices around the emulated cosmic string

To further the theoretical understanding of the bound vortices around the cosmic string, one could solve the Hamilton equation. Although there was not a general analytical solution for the equation, one could obtain an approximate solution as $\psi _e = \left( {r^{ - \frac{m}{\alpha }}e^{ - im\theta },r^{ - \frac{n}{\alpha }}e^{in\theta }} \right)^T$ with the angular momentum *m* and *n* near the origin of the cosmic strings (see details in the Supplementary Section I). The case of *m* ≤ 0 and *n* ≤ 0 corresponded to a radiating mode with the increasing intensity $I = \left| {\psi _e\psi _e^ \ast } \right|$ for the large distance. In contrast, the case as *m* > 0 and *n* > 0 corresponded to a bound state with the decreasing intensity $I = \left| {\psi _e\psi _e^ \ast } \right|$ with the large distance, and the energy distribution was confined around the origin. Therefore, this nontrivial property could confine vortex modes with positive vortex charges. Additionally, this nontrivial property indicated that bound vortices circled around in a clockwise direction, but the reverse was not allowable (see the Supplementary videos). With the excitation of one single waveguide in the innermost ring layer around the cosmic string, one could assume that the excitation state was the superposition of the Dirac fermion with many different vortex charges. Although some energy radiated out, a large portion of energy was confined and twisted around the string with a clockwise direction.

**Fig. 3:**
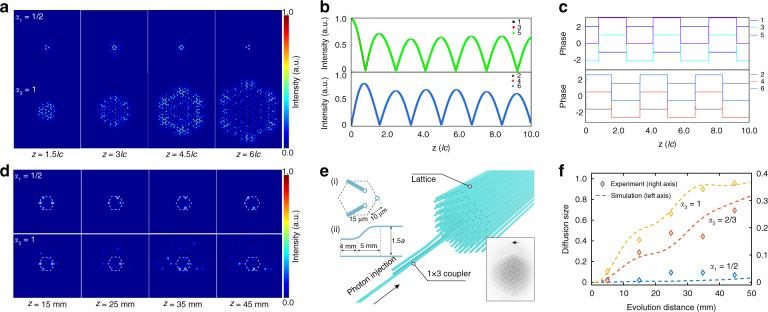
The bound capability of optical vortices in cosmic strings with gauge fields. **a** and **d** are respectively the theoretical calculated evolution and experimental results with different propagation distances in the cosmic string (*α*_1_ = 1/2) and flat space (*α*_3_ = 1). The white dashed hexagon line indicated the innermost ring site. **b** and **c** are respectively the intensity and phase distribution as the evolution among the six sites in the innermost ring layer. **e** is the experimental schematic for exciting a series of waveguides in the innermost ring layer around the cosmic string. **f** is the diffusion size as the evolution distance in the cosmic string and flat space

To clearly exhibit bound vortices for the gauge field of the cosmic string in experiments, we also fabricated a 1×3 coupler before the lattice and connected it to three sites with the same sublattice in the innermost layer, as shown in Fig. 3e. By exciting the input waveguide of the 1×3 coupler, we could the obtain initial state $\left| {\varphi _{in}} \right\rangle = \mathop {\sum}\nolimits_n {e^{i\theta _i}} \left| i \right\rangle$ as the vortex fermion mode, where *i* ranged from 1 to 3 as the site label, and *θ*_*i*_ was the phase magnitude for the *i*th site (see details in section IV in the Supplementary). By modulating the hopping and the on-site energy in the fabrication, we obtained the initial state $\left| {\varphi _{in}} \right\rangle$ with $\theta _1 \,\ne\, \theta _2 \,\ne\, \theta _3$ and injected it into the lattice. As shown in Fig. 3d, the optical vortex mode was well-bound for the gauge field of the cosmic string but not in the flat space. As illustrated in Fig. 3f, we compared the diffusion size for the evolution distance for different density parameters, which distinctly showed that the emulated cosmic string with a smaller density parameter, corresponding to a larger magnitude of the gauge field, had a stronger confinement capability.

**Fig. 4:**
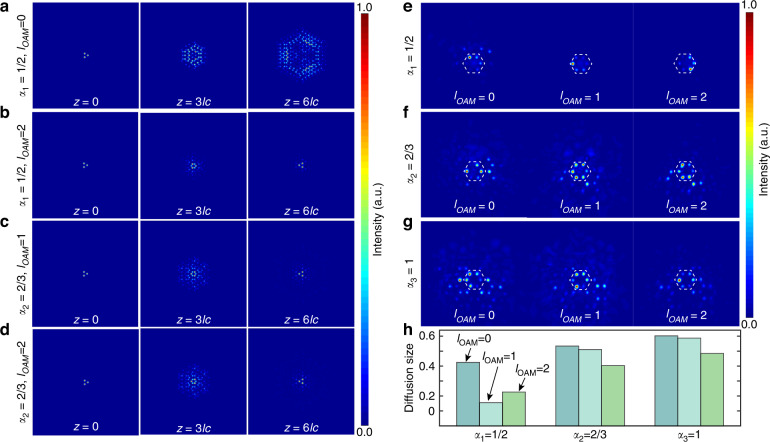
The ability to distinguish photons whether they had orbital angular momentum or not. **a** and **b** are respectively the theoretical calculated results for light without orbital angular momentums and with orbital angular momentum *l*_*OAM*_ = 2 in the cosmic string with mass density parameter *α*_1_ = 1/2. **c** and **d** are respectively the theoretical calculated results for light with orbital angular momentum *l*_*OAM*_ = 1,2 in the cosmic string with mass density parameter *α*_2_ = 2/3. **e**, **f** and **g** are the experiment results of light with and without angular momentums in the cosmic string with different mass density parameter (*α*_1_ = 1/2, *α*_2_ = 2/3, *α*_3_ = 1). The white dashed hexagon line indicated the innermost ring site. **h** is the comparison of the diffusion size for light with different angular momentums in the flat space and in the cosmic string

Furthermore, this type of deformed photonic graphene inspired by cosmic strings has the capability of distinguishing photons with or without orbital angular momentum. Unlike photons with orbital angular momentums, when the electromagnetic waves without orbital angular momentum (*l*_*OAM*_ = 0) were injected into this type of deformed photonic graphene, the radiation modes were excited and diffused to all sites in the lattice, as shown in Fig. 4a, which was the natural result of the wave dynamics near the Dirac point (see Fig. S1 in Supplementary). We directly injected the vortex beams with different charges, such as, *l*_*OAM*_=0, *l*_*OAM*_ = 1 and *l*_*OAM*_ = 2, into the deformed photonic graphene lattices from the innermost ring layer in the experiments. Then we experimentally measured the evolution results for the same propagation (50 mm), as shown in Fig. 4e–g. Figure 4h shows the quantified diffusion size for different cases for different orbital angular momentums and the gauge fields of cosmic strings. These results clarified the fact that the light with orbital angular momentum in the emulated large gauge field of cosmic strings exhibited a well-defined local confinement, whereas the light spread for the case without orbital angular momentum. Hence, the deformed photonic graphene had the capability of distinguishing photons with or without orbital angular momentum. While the current deformed photonic graphene cannot distinguish different charges of angular momentums just as shown in Fig. 4c and d that indicates the evolution patterns are almost same with inputting different angular momentums. In the following work, we will change the design of the deformed photonic graphene to have the capability for distinguishing photons not only whether with angular momentums but also with the charge of angular momentums.

## Discussion

In summary, we exploited a deformed photonic graphene lattice to simulate a gauge field of topological cosmic string. The deformed photonic lattice inspired by topological cosmic strings could generate and transport optical bound vortices at the same time, and even manipulate the angular momentum of photons on a chip. This novel feature may have potential applications on integrated photonic chip. In this work, we just emulated the gauge field of cosmic strings through effective Dirac Hamiltonian in a deformed photonic graphene. Whereas other issues of cosmic strings, such as their creation and dynamics during early-time evolution of the universe, may be mapped into another type of photonic lattices. Additionally, the design of bound states can also be utilized to enhance nonlinearity of optical fibers, which may make them as quantum light source in photonic computing. Furthermore, the developed experimental platform of photonic lattices also has a potential in neuromorphic quantum computer^67,68^ inspired by the mechanism of memristor.

## Materials and methods

We fabricate the designed photonic lattice using the femtosecond laser direct writing technology. We fabricate the samples in borosilicate glass substrate (refractive index *n*_0_ = 1.514 for the writing laser) using the femtosecond laser system operating at a wavelength of 513 nm, a repetition rate of 1 MHz and a pulse duration of 290 fs. The light is reshaped with a cylindrical lens and then is focused inside the sample with a 50× microscope objective (NA = 0.55). The substrates are continuously moved using a high-precision three-axis translation stage with a constant velocity of 10 mm/s, and the lattices are created by the laser-induced refractive index increase. For each a waveguide in the photonic lattice, it supports a single-mode waveguide for the propagation of wavelength at 810 nm. And the characterized relationship of the coupling coefficients and the separation between adjacent waveguides satisfies the formula as *t* = 3.30 *e*^*−*0.19*d*^. And *d* is the distance between adjacent waveguide sites. According to such a relation, we arrange the distance of waveguide site in a deformed photonic graphene to realize the designed anisotropic and nonuniform coupling coefficients required by emulating such a topological linear defect.

## Supplementary information

Bound Vortex Light in an Emulated Topological Defect in Photonic Lattices

Movies S1

Movies S2

Movies S3

Movies S4
