# Supplementary material for: Bound vortex light in an emulated topological defect in photonic lattices
Source: Light Sci Appl. 2022 Aug 1;11:243. doi: 10.1038/s41377-022-00931-4 (PMC9343378; doi:10.1038/s41377-022-00931-4)
Supplement: Supplementary file 1 — Bound Vortex Light in an Emulated Topological Defect in Photonic Lattices [file 41377_2022_931_MOESM1_ESM.pdf]

Supplementary Information for

Bound Vortex Light in an Emulated Topological Defect in Photonic Lattices

Chong Sheng[1]†, Yao Wang[2]†, Yijun Chang[2], Huiming Wang[2], Yongheng Lu[2], Yingyue Yang[2], Shining Zhu[1], Xianmin Jin[2,3]*, and Hui Liu[1]*

[1]National Laboratory of Solid State Microstructures and School of Physics, Collaborative Innovation Center of Advanced Microstructures, Nanjing University, Nanjing, Jiangsu 210093, China.

[2] Center for Integrated Quantum Information Technologies (IQIT), School of Physics and Astronomy and State Key Laboratory of Advanced Optical Communication Systems and Networks, Shanghai Jiao Tong University, Shanghai 200240, China

[3]CAS Center for Excellence and Synergetic Innovation Center in Quantum Information and Quantum Physics, University of Science and Technology of China, Hefei 230026, China

*E-mail: xianmin.jin@sjtu.edu.cn

*E-mail: liuhui@nju.edu.cn

†Equally contributed to this work.

I) The Dirac equation under a gauge field of the cosmic string

We start by considering the line element of a spacetime metric for a static cylindrically symmetric cosmic string at a certain plane in the low-mass limit with straight and infinite long length:

$$ds^2 = -dt^2 + dr^2 + \alpha^2 r^2 d\theta^2 \tag{S1}$$

We obtain the element of metric as

$$g_{tt} = -1, g_{rr} = 1, g_{\theta\theta} = \alpha^2 r^2, g^{tt} = -1, g^{rr} = 1, g^{\theta\theta} = 1/(\alpha^2 r^2) \tag{S2}$$

Then we can get the calculated Christoffel symbol as

$$\Gamma^i_{kl} = \frac{1}{2} g^{im} \left(\frac{\partial g_{mk}}{\partial x^l} + \frac{\partial g_{ml}}{\partial x^k} - \frac{\partial g_{kl}}{\partial x^m} \right) \tag{S3}$$

The Einstein convention is adopted here where the repeated indices are summed over. We have for nonzero Christoffel symbol as

$$\Gamma^\theta_{r\theta} = \Gamma^\theta_{\theta r} = 1/r \, , \Gamma^r_{\theta\theta} = -\alpha^2 r \tag{S4}$$

The calculated spinor connection ω^a_{bv} can be directly obtained as

$$\omega^a_{bv} = e^a_u \partial_v (e^u_b) + e^a_u e^\sigma_b \Gamma^u_{\sigma v} \tag{S5}$$

where e_u^a (we use the convention that latin indices a, b are used to label local inertial coordinates and greek indices u, v for general coordinates) is *vielbein* and satisfy the equation $e_a^u e_b^v g_{uv} = \eta_{ab}$, $e_u^a e_v^b \eta_{ab} = g_{uv}$, where $\eta_{ab} = \mathrm{diag}(-1,1,1)$ is the Minkowski metric. Here we choose *vielbein* as:

$$e_u^a = \begin{bmatrix} cos\theta & -\alpha r sin\theta \\ sin\theta & \alpha r cos\theta \end{bmatrix}, \quad e_a^u = \begin{bmatrix} cos\theta & sin\theta \\ -sin\theta/(\alpha r) & cos\theta/(\alpha r) \end{bmatrix} \tag{S6}$$

Based on Eq.(S5), the nonzero spinor connection is

$$\omega_{r\theta\theta} = -\omega_{\theta r\theta} = 1-\alpha \tag{S7}$$

Using the spin connection, we can obtain the calculated spinor $\Omega_v = \frac{1}{4}\omega_{abv}\sigma^{ab}$, where $\sigma^{ab} = [\gamma^a, \gamma^b]/2$, and $\gamma^{a(b)}$ is Pauli matrix. Then nonzero spinor is

$$\Omega_\theta = \frac{1}{2}(1-\alpha)\sigma^{12} = \frac{1}{4}(1-\alpha)[\gamma^1, \gamma^2] \tag{S8}$$

For the massless Dirac equation with a gauge field in the presence of cosmic strings as follows:

$$\gamma^u(\partial_u + \Omega_u + iA_u)\psi = 0 \tag{S9}$$

with γ^u being Dirac matrix in a curved background, Ω_u being spin connection, and A_u being gauge field. After considering the gauge field $A_x = \eta \sin 2\theta$, $A_y = \eta \cos 2\theta$ with $\eta = -(1-\alpha)/(1+\alpha)$ in the flat space, the corresponding gauge field in the spacetime of cosmic strings as:

$$A_u = \frac{\partial x^a}{\partial x^u} A_a \tag{S10}$$

Hence, we obtain

$$A_t = 0, A_r = \frac{\partial x}{\partial r}A_x + \frac{\partial y}{\partial r}A_y = \eta \sin 3\theta, A_\theta = \frac{\partial x}{\partial \theta}A_x + \frac{\partial y}{\partial \theta}A_y = \alpha r \eta \cos 3\theta \tag{S11}$$

Owing to $\nabla_u = \partial_u + \Omega_u$, $\gamma^u = e_a^u \gamma^a$, Eq.(S9) can be written as:

$$\gamma^t \partial_t \psi + \gamma^r(\partial_r \psi + iA_r) + \gamma^\theta(\partial_\theta + \Omega_\theta + iA_\theta)\psi = 0 \tag{S12}$$

We choose $\gamma^0 = i\sigma_z, \gamma^1 = \sigma_y, \gamma^2 = -\sigma_x$, then $\Omega_\theta = \frac{1}{4}(1-\alpha)[\gamma^1, \gamma^2] = \frac{i}{2}(1-\alpha)\sigma_z, \gamma^t = -\gamma^0 = -i\sigma_z, \gamma^r = e_a^r \gamma^a = \cos\theta\,\sigma_y - \sin\theta\,\sigma_x, \gamma^\theta = e_a^\theta \gamma^a = \frac{1}{\alpha r}(-\sin\theta\,\sigma_y - \cos\theta\,\sigma_x)$. After adopting *ansatz* the solution $\psi = \frac{1}{\sqrt{r}} S(\theta)\psi_e = \frac{1}{\sqrt{r}} exp\left(-i\frac{\theta\sigma_z}{2}\right)\psi_e$, we obtain the Hamilton as:

$$i\partial_t\psi_e = -i\sigma_x(\partial_r\psi_e + i\eta\, sin\, 3\,\theta\psi_e) - \frac{1}{\alpha r} i\sigma_y(\partial_\theta\psi_e + i\alpha r\eta\, cos\, 3\,\theta\psi_e) \qquad (S13)$$

The equation (S13) describes the Dirac Fermion with anisotropic velocity in polar coordinates as $v_r/v_\theta = \alpha$, and the magnetic field under the presence of the cosmic string as:

$$\vec{B} = \nabla \times \vec{A} =$$

$$\begin{vmatrix} \vec{e}_x & \vec{e}_y & \vec{e}_z \\ \partial_x & \partial_y & \partial_z \\ \eta \sin 2\,\theta & \eta \cos 2\,\theta & 0 \end{vmatrix} = -2\eta\left(\frac{\partial\theta}{\partial x}\sin 2\,\theta + \frac{\partial\theta}{\partial y}\cos 2\,\theta\right)\vec{e}_z = -\frac{2\eta}{r}\cos 3\,\theta\vec{e}_z \qquad (S14)$$

To find the solution of Eq.(S13), we take the *ansatz* $\psi_e = \binom{a}{b} e^{-iEt}$ and obtain the equations:

$$-i\partial_r b - \partial_\theta b/(\alpha r) - i\eta e^{i3\theta} b = Ea$$

$$-i\partial_r a - \partial_\theta a/(\alpha r) - i\eta e^{i3\theta} a = Eb \qquad (S15)$$

Although there is not existing general analytical solution for Eq.(S15), we can achieve special solution after some approximations:

1) When considering $r \to 0$, Eq.(S15) can be written as:

$$-i\partial_r b - \partial_\theta b/(\alpha r) = 0, \qquad -i\partial_r a + \partial_\theta a/(\alpha r) = 0 \qquad (S16)$$

Considering $a = A(r)e^{-im\theta}$, $b = B(r)e^{in\theta}$, we obtain the analytic solution:

$$\psi_e = \begin{pmatrix} r^{-m/\alpha} e^{-im\theta} \\ r^{-n/\alpha} e^{in\theta} \end{pmatrix} e^{-iEt} \qquad (S17)$$

2) At the Dirac point $E = 0$, the coupled equations of Eq.(S15) become decoupled and can be written as:

$$-i\partial_r b - \partial_\theta b/(\alpha r) - i\eta e^{i3\theta} b = 0, \qquad -i\partial_r a - \partial_\theta a/(\alpha r) - i\eta e^{i3\theta} a = 0 \qquad (S18)$$

we obtain the analytic solution as:

$$\psi_e = \begin{pmatrix} c_1 exp\left(\alpha\eta r e^{-3i\theta}/(3+\alpha)\right) \\ c_2 exp\left(-\alpha\eta r e^{3i\theta}/(3+\alpha)\right) \end{pmatrix} \qquad (S19)$$

The radiation mode according to theoretical calculation respectively based on Eq.(S19) and the deformed optical graphene lattice just as shown in Fig. S1 are good agreement.

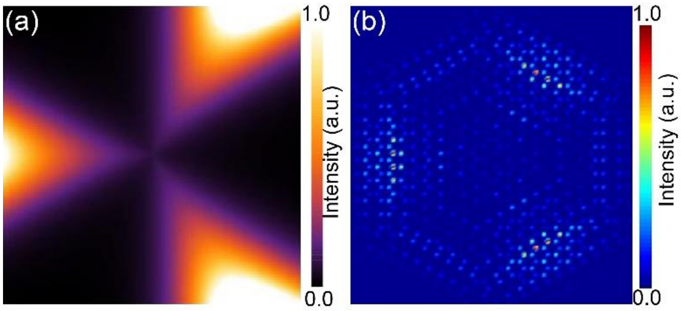

Fig. S1 (a) The radiating mode near the Dirac point numerically calculated based on Eq.(S19). (b) The radiating mode in the deformed optical graphene lattice.

II) The gauge field constructed by a deformed photonic graphene

We are interested in the low-energy Hamiltonian, *i.e*, the effective Dirac Hamiltonian, of a honeycomb lattice with anisotropy in the nearest-neighbor hopping parameters. As unstrained graphene, our lattice consists of a triangular Bravais lattice with a pair of atoms (open and filled circles in Fig. S2(a)) located in its primitive cell. However, we consider that the hopping between nearest sites are space dependent and, in general, are characterized by three hopping parameters t_1,t_2,t_3. Within this nearest-neighbor tight-binding model, one can demonstrate that the Hamiltonian in momentum space can be represented by a 2×2 matrix of the form

$$H = -\sum_{n=1}^{3} t_n \begin{pmatrix} 0 & e^{-i\vec{k}\cdot\vec{\delta}_n} \\ e^{i\vec{k}\cdot\vec{\delta}_n} & 0 \end{pmatrix} \qquad (S20)$$

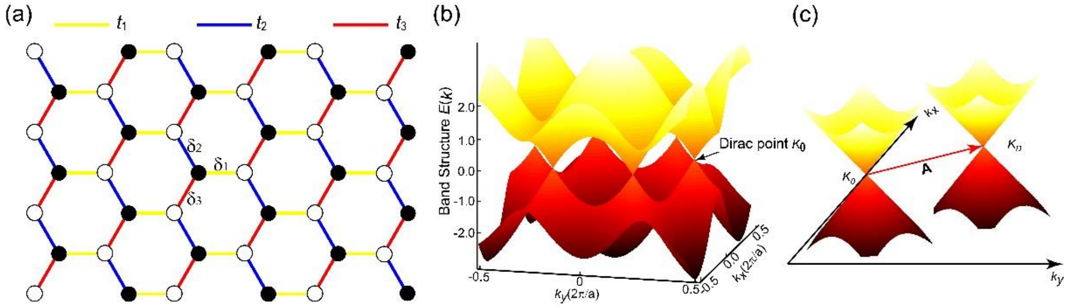

Fig. S2 (a) Honeycomb lattice and hoppings. The primitive cell contains a pair of atoms. (b) Dispersion relation $E(k)$ of the anisotropic honeycomb lattice and its hexagonal first Brillouin zone. (c) A zoom of the Dirac cone region shows that the Dirac point K_D is not located at the corner K_0 of the hexagonal Brillouin zone. The anisotropy-induced Dirac point shift is given by the vector $\boldsymbol{A}$.

where δ_1, δ_2, δ_3 are the nearest-neighbor vectors, and are respectively defined as $\vec{\delta}_1 = a(1,0)$, $\vec{\delta}_2 = \frac{a}{2}(-1,\sqrt{3})$, $\vec{\delta}_3 = \frac{a}{2}(-1,-\sqrt{3})$. From Eq.(S20) follows that the dispersion relation is given by two bands,

$$E(\vec{k}) = \pm\left|t_1 e^{-i\vec{k}\cdot\vec{\delta}_1} + t_2 e^{-i\vec{k}\cdot\vec{\delta}_2} + t_2 e^{-i\vec{k}\cdot\vec{\delta}_3}\right| \qquad (S21)$$

As is well document, for the isotropic case $t_1 = t_2 = t_3 = t_0$, the Dirac points $\vec{K}_D$, which are determined by condition $E(\vec{K}_D) = 0$, coincide with the corners of the first Brillouin zone. Then, to obtain the Dirac Hamiltonian in this case, one can simply expand the Hamiltonian (S20) around a corner, e.g. $K_0 = \left(0, 4\pi/(3\sqrt{3}a)\right)$ (just as shown in Fig. S2(b)). However, for the considered anisotropic case[1] $t_n = t_0(1 + \Delta_n)$, the Dirac point K_D do not coincide with the corners of the first Brillouin zone as shown in Fig. S1(c). The condition $E(\vec{K}_D) = 0$, can be equivalently rewritten as

$$\sum_{n=1}^{3} t_n e^{i\vec{K}_D\cdot\vec{\delta}_n} = 0 \qquad \text{(S22)}$$

Then, one can propose the position of $\vec{K}_D$ in the form

$$\vec{K}_D = \vec{K}_0 + \vec{A} + O(\Delta_n^2) \qquad \text{(S23)}$$

where the unknown shift $\vec{A}$ will be looked for as a linear combination on the parameters $\{\Delta_n\}$. Now, substituting Eq.(S23) into Eq.(S22) results in

$$\sum_{n=1}^{3} t_0(1 + \Delta_n)e^{i[\vec{K}_0+\vec{A}+O(\Delta_n^2)\ ]\cdot\vec{\delta}_n} = 0$$

$$\sum_{n=1}^{3} t_0(1 + \Delta_n)\left(1 + i\vec{A}\cdot\vec{\delta}_n\right)e^{i\vec{K}_0\cdot\vec{\delta}_n} = 0$$

$$\sum_{n=1}^{3} t_0\left(1 + \Delta_n + i\vec{A}\cdot\vec{\delta}_n + O(\Delta_n^2)\right)e^{i\vec{K}_0\cdot\vec{\delta}_n} = 0$$

$$\Delta_1 - \frac{\Delta_2 + \Delta_3}{2} - \frac{3}{2}A_y a + i\frac{3A_x a}{2} + i\frac{\sqrt{3}(\Delta_2 - \Delta_3)}{2} = 0 \qquad \text{(S24)}$$

Thus, one obtains that the shift $\vec{A}$ is given by

$$A_x = \frac{1}{\sqrt{3}a}(\Delta_3 - \Delta_2), \quad A_y = \frac{1}{3a}(2\Delta_1 - \Delta_2 - \Delta_3) \qquad \text{(S25)}$$

Furthermore, the effective Dirac Hamiltonian around Dirac point $\vec{K}_D$ by considering momenta close to the Dirac point $\vec{K}_D$, i.e., $\vec{k} = \vec{K}_D + \vec{q}$, and expanding to first order $\vec{q}$ and $\{\Delta_n\}$, Hamiltonian (S20) transform as

$$H = -\sum_{n=1}^{3} t_n \begin{pmatrix} 0 & e^{-i(\vec{K}_0+\vec{A}+\vec{q})\cdot\vec{\delta}_n} \\ e^{i(\vec{K}_0+\vec{A}+\vec{q})\cdot\vec{\delta}_n} & 0 \end{pmatrix}$$

$$= -\sum_{n=1}^{3} t_0(1+\Delta_n)\begin{pmatrix} 0 & e^{-i\vec{K}_0\cdot\vec{\delta}_n} \\ e^{i\vec{K}_0\cdot\vec{\delta}_n} & 0 \end{pmatrix}\left(\bar{I} + i\sigma_z\vec{A}\cdot\vec{\delta}_n\right)\left(\bar{I} + i\sigma_z\vec{q}\cdot\vec{\delta}_n\right)$$

$$= -\sum_{n=1}^{3} t_0\frac{\vec{\sigma}\cdot\vec{\delta}_n}{a}\left[\bar{I} + i\sigma_z\vec{q}\cdot\vec{\delta}_n + i\sigma_z\vec{A}\cdot\vec{\delta}_n + \Delta_n\bar{I} - (\vec{q}\cdot\vec{\delta}_n)(\vec{A}\cdot\vec{\delta}_n)\bar{I}\right] \qquad \text{(S26)}$$

Collecting the contribution of each term in this expression, one obtains:

$$-t_0\sum_{n=1}^{3} t_0\frac{\vec{\sigma}\cdot\vec{\delta}_n}{a} = 0$$

$$-\sum_{n=1}^{3} t_0\left(\frac{\vec{\sigma}\cdot\vec{\delta}_n}{a}\right)\left(i\sigma_z\vec{q}\cdot\vec{\delta}_n\right) = i\sigma_z\hbar v_F\vec{\sigma}\cdot\vec{q}$$

$$-\sum_{n=1}^{3} t_0\left(\frac{\vec{\sigma}\cdot\vec{\delta}_n}{a}\right)\left(i\sigma_z\vec{A}\cdot\vec{\delta}_n\right) = i\sigma_z\hbar v_F\vec{\sigma}\cdot\vec{A}$$

$$-\sum_{n=1}^{3} t_0\frac{\vec{\sigma}\cdot\vec{\delta}_n}{a}\,\Delta_n\bar{I} = -i\sigma_z\hbar v_F\vec{\sigma}\cdot\vec{A}$$

$$\textstyle\sum_{n=1}^{3} t_0\left(\frac{\vec{\sigma}\cdot\vec{\delta}_n}{a}\right)(\vec{q}\cdot\vec{\delta}_n)(\vec{A}\cdot\vec{\delta}_n) = i\sigma_z\hbar v_F\vec{\sigma}\cdot\vec{\Delta}\cdot\vec{q} \qquad \text{(S27)}$$

where $v_F = \frac{3a_0t_0}{2\hbar}$, $\vec{\Delta}=\begin{pmatrix} \mathit{\Delta}_1 & \frac{1}{\sqrt{3}}(\mathit{\Delta}_3-\mathit{\Delta}_2) \\ \frac{1}{\sqrt{3}}(\mathit{\Delta}_3-\mathit{\Delta}_2) & \frac{1}{3}(2\mathit{\Delta}_2+2\mathit{\Delta}_3-\mathit{\Delta}_1) \end{pmatrix}$. Then, considering the contribution of each term in Eq.(S26), the effective Dirac Hamiltonian around $\overrightarrow{K_D}$ has the form:

$$E_F = \hbar v_F i\sigma_z\vec{\sigma}\cdot\left(\begin{pmatrix} 1 & 0 \\ 0 & 1 \end{pmatrix} + \begin{pmatrix} \mathit{\Delta}_1 & \frac{1}{\sqrt{3}}(\mathit{\Delta}_3-\mathit{\Delta}_2) \\ \frac{1}{\sqrt{3}}(\mathit{\Delta}_3-\mathit{\Delta}_2) & \frac{1}{3}(2\mathit{\Delta}_2+2\mathit{\Delta}_3-\mathit{\Delta}_1) \end{pmatrix}\right)\cdot\vec{q} \qquad \text{(S28)}$$

In order to obtain Dirac fermion with anisotropic velocity in polar coordinates, one can take perturbed hopping parameters as $\mathit{\Delta}_1 = \eta\cos 2\theta, \mathit{\Delta}_2 = -\eta\cos\left(2\theta-\frac{\pi}{3}\right), \mathit{\Delta}_3 = -\eta\cos\left(2\theta+\frac{\pi}{3}\right)$, and obtain effective Dirac Hamiltonian

$$E_F = \hbar v_F i\sigma_z\vec{\sigma}\cdot\left(\begin{pmatrix} 1 & 0 \\ 0 & 1 \end{pmatrix} + \begin{pmatrix} \eta\cos 2\theta & \eta\sin 2\theta \\ \eta\sin 2\theta & -\eta\cos 2\theta \end{pmatrix}\right)\cdot\vec{q}$$

$$=\hbar v_F i\sigma_z\vec{\sigma}\cdot U^{-1}(\theta)\begin{pmatrix} 1+\eta & 0 \\ 0 & 1-\eta \end{pmatrix}U(\theta)\cdot\vec{q} \qquad \text{(S29)}$$

with $U(\theta) = \begin{pmatrix} \cos\theta & \sin\theta \\ -\sin\theta & \cos\theta \end{pmatrix}$ being rotate operator. Based on effective Dirac Hamiltonian, the anisotropic velocity of Dirac fermion in polar is taken as $\frac{v_r}{v_\theta} = \frac{(1+\eta)}{(1-\eta)} =$

α. One can also obtain the gauge field as $A_x = \eta \sin 2\theta, A_y = \eta \cos 2\theta$, which means that the momentum shift of Dirac cone ($\vec{K}_D$) circles around the unperturbed Dirac cone ($\vec{K}_0$) (just as shown in Fig. S2(c)). Finally, the quantum field under the gauge field in the presence of cosmic strings are achievable.

For K_0 with valley index $\xi = -1$, one can propose the position of $\vec{K}_D$ in the form $\vec{K}_D' = \vec{K}_0 - \vec{A}$. After considering the effective Dirac Hamiltonian around Dirac point $\vec{K}_D'$, the calculation is analogous and the effective Dirac Hamiltonian results,

$$E_F = \hbar v_F i \sigma_z \vec{\sigma}^* \cdot \left(\begin{pmatrix} 1 & 0 \\ 0 & 1 \end{pmatrix} + \begin{pmatrix} \Delta_1 & \frac{1}{\sqrt{3}}(\Delta_3 - \Delta_2) \\ \frac{1}{\sqrt{3}}(\Delta_3 - \Delta_2) & \frac{1}{3}(2\Delta_2 + 2\Delta_3 - \Delta_1) \end{pmatrix} \right) \cdot \vec{q}$$

where $\vec{\sigma}^* = (\sigma_x \quad -\sigma_y)$

Due to the similarities of the two Hamiltonians with different valley index, the evolution of different valleys has the same behavior. Fig. S3(a) and Fig. S3(b) respectively show the dynamic evolution with different propagation distances for exciting one of sublattice A and sublattice B in the inner layer around the cosmic string, which exemplifies that Dirac fermions with different valleys have the same evolution behavior.

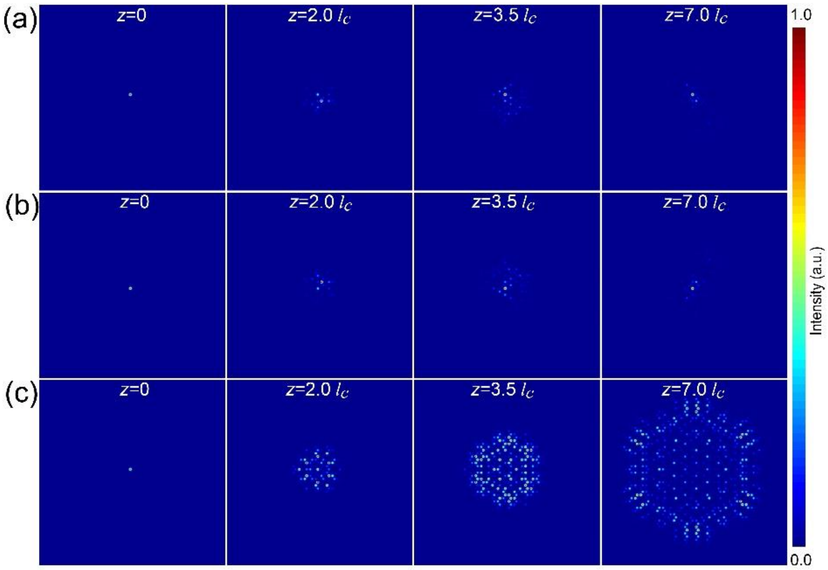

Fig. S3 (a) The dynamic evolution with different propagation distances for exciting one of sublattice A in the inner layer around the cosmic string; (b) the dynamic evolution with different propagation distances for exciting one of sublattice B in the inner layer around the cosmic string; (c) the dynamic evolution in uniform optical graphene for exciting a single waveguide.

III) The tight-binding model of the photonic lattice to emulate cosmic string

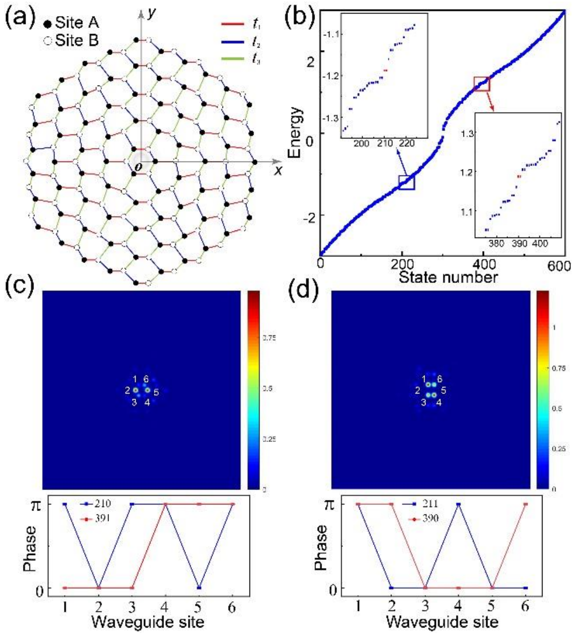

Fig. S4 (a) The deformed lattices to emulate the cosmic string; (b) the energy spectrum of the deformed lattices using 600 waveguide sites. (c) the intensity distribution of eigen state number for $n = 210$ and $n = 391$; and the phase distribution for the innermost layer for the corresponding eigen states;(d) the intensity distribution of eigen state number for $n = 211$ and $n = 390$; and the phase distribution for the innermost layer for the corresponding eigen states.

To study the qualitative features of the photonic lattice to emulate cosmic string, we calculate the energy spectrum of the corresponding tight-binding models. Fig. S4(a) shows the deformed photonic graphene lattice with a position-dependent coefficient to emulate the cosmic string with a gauge field. We convert the lattices into tight-binding Hamiltonians with nearest neighbor hopping as $t_1 = t_0(1 + \eta \, cos\, 2\,\theta)$, $t_2 = t_0\left(1 - \eta \, cos\left(2\theta - \frac{\pi}{3}\right)\right)$, $t_3 = t_0\left(1 - \eta \, cos\left(2\theta + \frac{\pi}{3}\right)\right)$ (Here $t_0 = 1$ is chosen for convenience, not to fit experiments). Fig. S4(b) shows energy spectrum of the deformed lattice with $\eta = -1/3$, which indicates the cosmic string with the density parameter $\alpha = 1/2$. After numerical calculations, we find there exist two pairs of special degenerate energies $E = \pm E_0$ as shown in the inset of Fig. S4(b). The eigen state number $n = 210$ ($n = 211$) and $n = 391$ ($n = 390$), which respectively correspond to the energy spectrum of E_0 and $-E_0$, have same intensity distribution. And the most energy is confined to the innermost layer of the deformed lattices as shown in Fig. S4(c) and Fig. S4(d). At the same time, from the phase distribution of the main intensity of the innermost layer sites, the eigen state number $n = 210$ with $E = -E_0$ is a symmetric mode, while the eigen state number $n = 390$ with $E = E_0$ is an antisymmetric mode.

IV) The evolution of vortex state around the cosmic string.

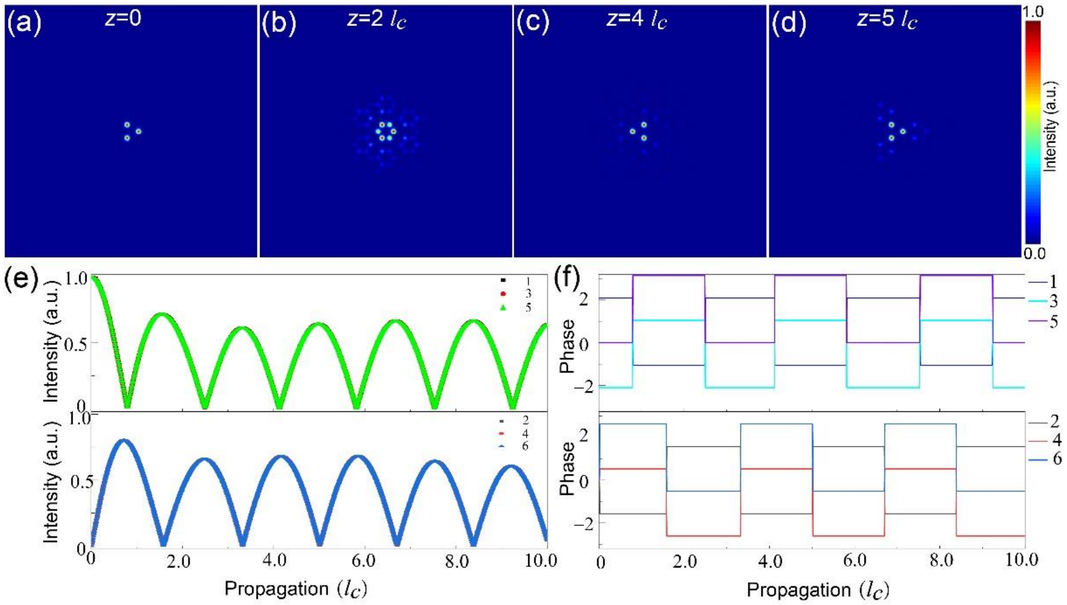

Fig. S5 (a)-(d) The evolution of exciting type A sites in the innermost layer of the deformed lattice; (e) the intensity oscillation between type A (number 1,3,5 site) and type B (number 2,4,6 site) sites as propagation; (f) the phase evolution of type A and type B sites.

Based on the analysis of eigen states, there are bound states in the innermost layer of the deformed lattice which emulate a comic string. If we excite the same type site of the innermost layer with a vortex state with an angular momentum (here we take $l = 1$ as an example just as shown in Fig. S5(a)-(d)), we find that there is an oscillation between type A sites and type B sites with a vortex just as shown Fig. S5(e). Fig. S5(f) clearly the phase difference between sites of the same type is always constant $\theta_0 = \pi/3$ with propagation, which means that such a vortex can stably propagate in such a deformed lattice.

Furthermore, if we excite all the sites of the innermost layer of such a lattice with an angular momentum (here we also take $l = 1$ as an example), we find that although some energy spreads out, most energy are confined in the innermost layer with the same phase difference $\theta_1 = \pi/6$, just as shown in Fig. S6(e) and (f). When the light propagates some enough distance, the intensity of the same type is constant during propagation, while there is a little discrepancy between the different type A (site 1,3,5) and type B site (site 2,4,6). Nevertheless, the vortex light can be efficiently bound around the origin of the deformed lattice to emulate cosmic string.

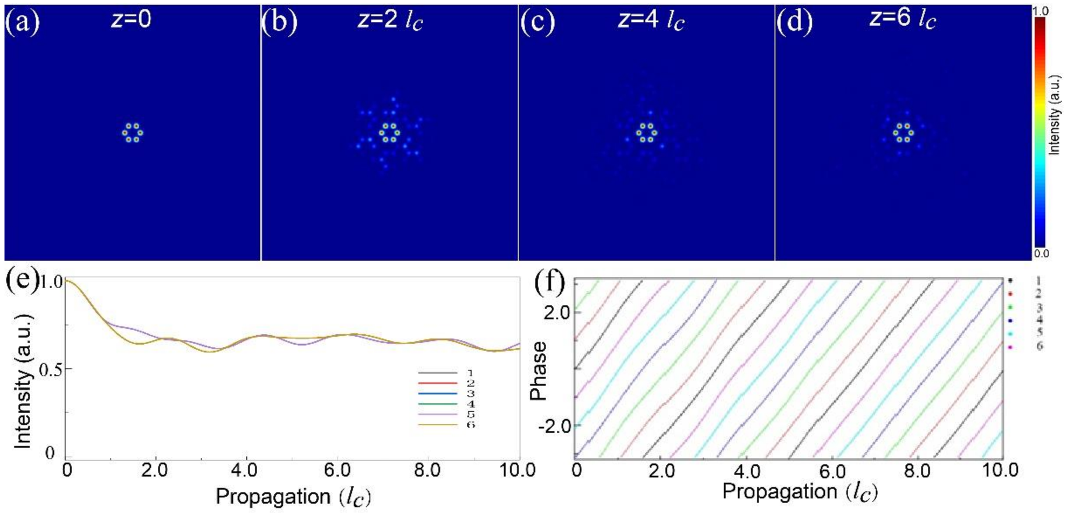

Fig. S6 (a)-(d) The evolution of exciting all sites in the innermost layer of the deformed lattice; (e) the intensity evolution of all sites; (f) the phase evolution of all sites.

V) The discussion between the curvature of cosmic strings and the exist of bound vortices

Considering the line element of cosmic-string space time as Eq.S1, the corresponding Riemann curvature is given by $R_{r,\theta}^{r,\theta} = \frac{1-\alpha}{4\alpha}\delta(r)$, where $\delta(r)$ is the Dirac delta function in the plane. Therefore, the string is locally flat except a conical singularity at the origin. According to Riemann curvature, if the mass density parameter $\alpha < 1$, it carries positive curvature at the origin; for the case $\alpha = 1$, it corresponds to the flat space; while it corresponds to an anticonical space time with negative curvature for $\alpha > 1$. Intriguingly, we found that there existed bound vortices only for the positive curvature at the origin of strings. Fig. S7 compares the theoretical calculated evolution of angular momentum lights as $l_{OAM} = 1$ with different propagation distances in the cosmic string with positive and negative curvature and flat space. We can clearly see that there exist bound vortices for mass density parameter $\alpha < 1$; while the angular momentum light with $l_{OAM} = 1$ cannot be confined for the case with $\alpha \geq 1$.

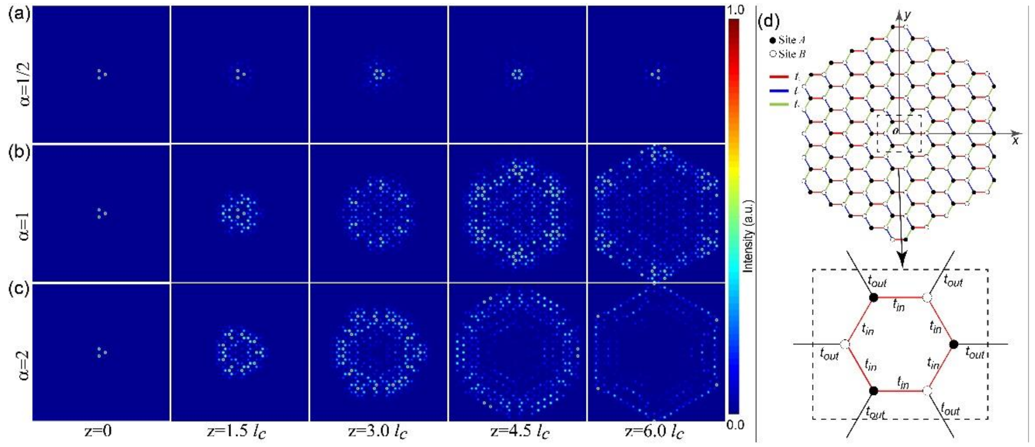

Fig. S7 (a)-(c) The evolution of angular momentum light with different propagation distances in the cosmic string under the density parameter $\alpha = 1/2$, $\alpha = 1$, $\alpha = 2$. (d) The comparison of coupling coefficients of waveguide in the innermost ring layer with that between the innermost layer and adjacent out layer.

To qualitatively analyze the relation about the existence of a bound vortex mode with the density parameter α, we can compare the coupling coefficient in the innermost ring layer with that between the innermost layer and the adjacent out layer (see Fig. S7(d)). The underlie reason is that for such a bound vortex mode, most energy are confined in the innermost ring layer. After considering the designed coupling coefficient, we find that the waveguides between the innermost ring layer have the same coupling coefficient dubbed as t_{in}, while for the case of the innermost ring layer between adjacent out layer they also have the same coupling coefficient dubbed as t_{out}. When the density parameter $\alpha = 1$ leading to $t_{in} = t_{out}$, there obviously exist no bound vortex light. For the case of $\alpha < 1$, we find that this result of $t_{in} > t_{out}$, which means that photons tend to couple among these waveguides in the innermost ring layer. Whereas for the case of $\alpha > 1$, it has the corresponding result of $t_{in} < t_{out}$, resulting in that photons tend to escape from the innermost ring layer.

VI) The discussion on the details in experiment

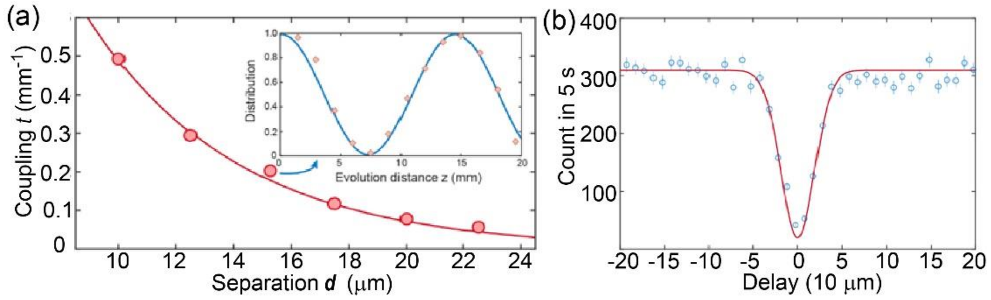

Fig. S8 (a) The relationship between the coupling coefficients and the separation between adjacent waveguides, the inset shown the distribution evolution for waveguide separated 15 μm; (b) The coincidence counts for indistinguishable photons after a 3D 2×2 photonic beam splitter.

We fabricate the designed photonic lattice using the femtosecond laser direct writing technology. According to the characterized relationship between the coupling coefficients and the separation between adjacent waveguides, as Fig. S8(a) shown, we fabricate the samples in borosilicate glass substrate (refractive index n_0=1.514 for the writing laser) using the femtosecond laser system operating at a wavelength of 513 nm, a repetition rate of 1 MHz and a pulse duration of 290 fs. The light is reshaped with a

cylindrical lens and then is focused inside the sample with a 50× microscope objective (NA=0.55). The substrates are continuously moved using a high-precision three-axis translation stage with a constant velocity of 10 mm/s, and the lattices are created by the laser-induced refractive index increase.

In experiment, we firstly characterize the coupling between two adjacent waveguides to calculate the coupling strength and demonstrate the stabilization of coupling process. As shown in Fig. S8(a), we inject the photon into one of the adjacent two waveguides, and measure dynamical intensity of photon in the excited waveguide, the measured result is cosine oscillation as shown in inset. From the photon dynamics between the adjacent waveguides, we can obtain the coupling strength, which depends on the separation space between the waveguides. To show the stabilization of coupling process, we implement the HOM interference for two indistinguishable photons after a 3D 2×2 photonic beam splitter (where the waveguides are not straight like in Fig. 3(e) of the main text). The HOM dip is successfully observed in the result shown in Fig. S8(b), which means the phase and polarization of photon are less influenced.

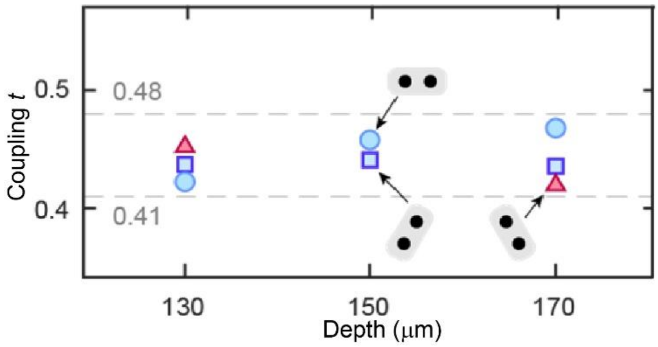

Fig. S9 The coupling strength between waveguides in deferent depths from the glass facet and in different directions.

As discussed above, the coupling strength is obtained from the fitting of photonic dynamics in adjacent waveguides, in which the error is naturally induced. This is one of the experimental imperfect sources. Besides, the designed lattice is 2D and the coupling direction is different. As shown in Fig. S9, we measure the coupling strength between waveguides in different depths from glass facet and in different directions. The coupling strengths keep in uniform with little coupling fluctuations, which contribute to the second experimental imperfect source. Such coupling fluctuations have little effect on the experimental results due to the limited propagational distance. Furthermore, we modulate the laser power to write waveguides in different depths to keep the uniform coupling and single-mode feature of waveguides, the discussed HOM

interference in 3D 2×2 photonic beam splitter above also demonstrates effect. Although the imperfections induced by fabrication and materials are unavoidable in experiments, the experimental results were agreed with the theoretical calculation and successfully demonstrated the ideas.

Apart from the imperfections, loss is also the point that should be concerned in experiments. In our experiment, though the laser-written waveguides are lossy, such lossy (0.2-0.3dB/cm in our experiment) is uniform for all the sites in the lattice, which will not influent the dynamics of photons in the deformed photonic graphene, especially we excite lattice with coherent photons.

In the process of measuring the photonic chip, the light is injected into lattice using a 20× objective lens, and the outgoing probability distributions of the photons outgoing from the chip are observed using a 10× microscope objective lens and a CCD camera, and the results are recorded in both images and corresponding raw digital data. The figures showing photon distributions in the main text, such as Fig. 2(b,d,g) and Fig. 3(d), are raw images after adjusting the color bar for appropriate visual results. The analyzed results, such as the diffusion size in Fig. 2(e) and bound index in Fig. 2(h), are obtained from the corresponding raw digital data.

VII) The experiment design of photonic lattice.

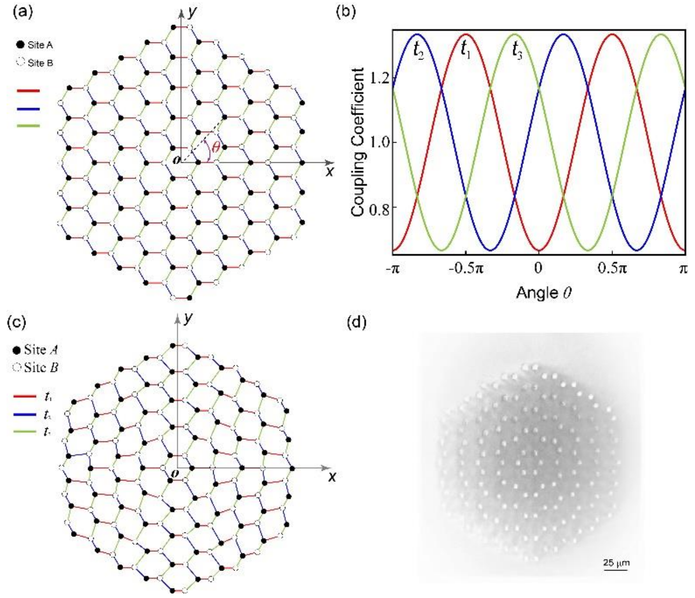

Fig. S10 (a) The ideal photonic graphene with an identical lattice distance but anisotropic coupling coefficients $t_{1,2,3}$. The symbol O is the origin center of the photonic graphene where the emulated topological linear defect locates. θ is the is azimuth angle of the waveguide site compared to the supposed location of the cosmic defect O. (b) The coupling efficient $t_{1,2,3}$ as a function of the azimuth angle θ. (c) The schematic of the deformed photonic graphene using the relationship

between the coupling coefficients and the separation distance between adjacent waveguides. (d) The experimental deformed photonic graphene.

According to the effective Hamiltonian after considering the massless Dirac equation in the presence of such a topological linear defect, we obtain anisotropic coupling coefficient as $t_1 = t_0(1 + \eta\, cos\, 2\,\theta)$, $t_2 = t_0\left(1 - \eta\, cos\left(2\theta - \frac{\pi}{3}\right)\right)$, $t_3 = t_0\left(1 - \eta\, cos\left(2\theta + \frac{\pi}{3}\right)\right)$ $(\eta = (\alpha - 1)/(\alpha + 1))$ in the ideal photonic lattice with the identical lattice distance. And θ is the is azimuth angle of the waveguide site compared to the supposed location of the cosmic defect, as shown in Fig. S10a. Considering the density parameter $\alpha = 1/2$, the controlling parameter in the coupling coefficient is $\eta = -1/3$. In experiment, we take $t_0 = 0.158$. And the characterized relationship between the coupling coefficients t and the separation distance between adjacent waveguides d satisfies the formula as $d = -\log(t/3.30)/0.19$. Fig. S10c shows the deformed photonic graphene lattice to satisfy the designed coupling coefficient required by the emulated cosmic string. And Fig. S10d is the experimental figure. Despite that the azimuth angle θ is not conserved, the original ideal photonic graphene and the deformed photonic graphene have the same equivalent Hamiltonian to depict the evolution of photons.

Reference

1 Oliva-Leyva, M. & Naumis, G. G. Effective Dirac Hamiltonian for anisotropic honeycomb lattices: Optical properties. *Phys.Rev. B* **93**, 035439 (2016).

Movies S1: This video exhibits that when a single waveguide in the innermost ring layer around the origin of the deformed photonic graphene, which emulates a cosmic string, is pumped, a large portion of energy was confined and twisted around the string with a clockwise direction.

Movies S2: This video exhibits that when a single waveguide in the uniform photonic graphene is pumped, there is no bound state and light is spreading outward.

Movies S3: This video exhibits that when three same type waveguide sites in the innermost ring layer around the origin of the deformed photonic graphene, which emulates a cosmic string, are pumped with the different phases ($\theta_1 = 0, \theta_2 = 2\pi/3, \theta_3 = 4\pi/3$), there is a vortex bound state circling around the origin of the cosmic string.

Movies S4: This video exhibits that when three same type waveguide sites in the uniform photonic graphene are pumped with the same phase, there exist only the radiating mode.
