# Supplementary figures and images for: Bound vortex light in an emulated topological defect in photonic lattices
Source: Light Sci Appl. 2022 Aug 1;11:243. doi: 10.1038/s41377-022-00931-4 (PMC9343378; doi:10.1038/s41377-022-00931-4)

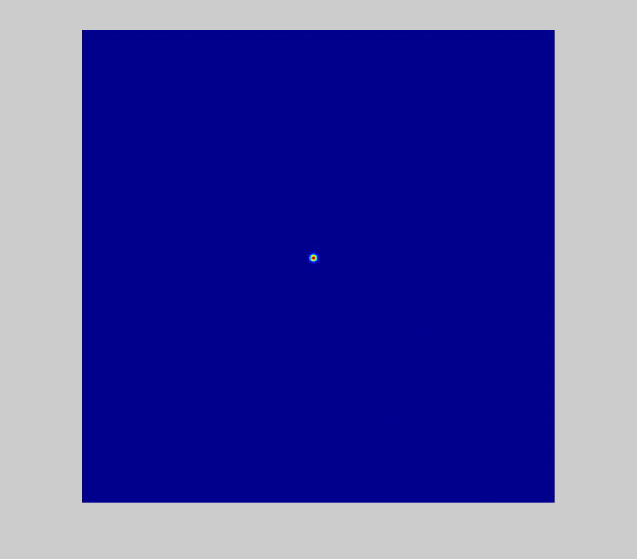

Supplement: Supplementary file 2 — Movies S1 [file 41377_2022_931_MOESM2_ESM.gif]

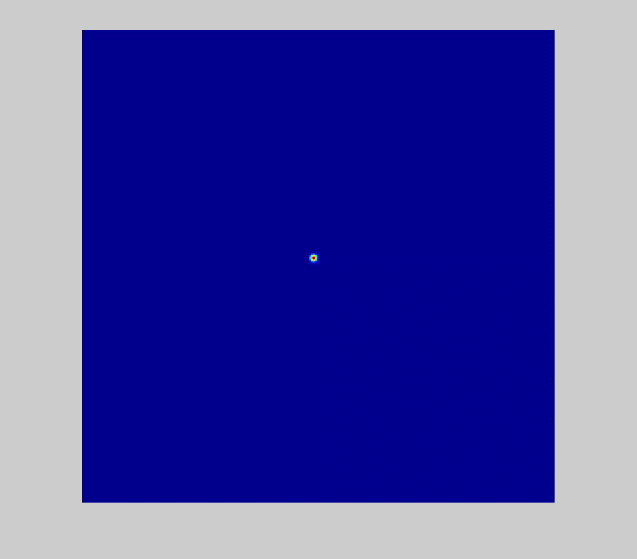

Supplement: Supplementary file 3 — Movies S2 [file 41377_2022_931_MOESM3_ESM.gif]

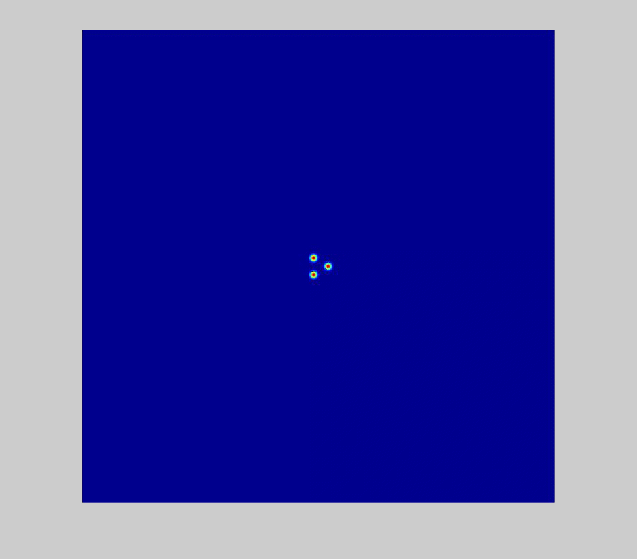

Supplement: Supplementary file 4 — Movies S3 [file 41377_2022_931_MOESM4_ESM.gif]

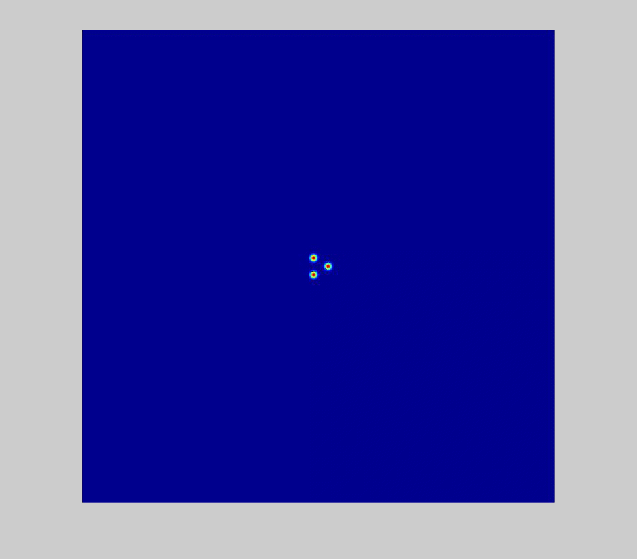

Supplement: Supplementary file 5 — Movies S4 [file 41377_2022_931_MOESM5_ESM.gif]
